# Dry Powder Inhalers for Delivery of Synthetic Biomolecules

**DOI:** 10.3390/ph18020175

**Published:** 2025-01-27

**Authors:** Hossein Omidian, Ali Nokhodchi, Niloofar Babanejad

**Affiliations:** 1 Barry and Juddy Silverman College of Pharmacy, Nova Southeastern University, Fort Lauderdale, FL 33328, USA; 2 Lupin Inhalation Research Center, 4006 NW 124th Ave, Coral Springs, FL 33065, USA; alinokhodchi@lupin.com; 3 CONRAD, Eastern Virginia Medical School, Norfolk, VA 23507, USA

**Keywords:** dry powder inhalers, synthetic biomolecules, pulmonary drug delivery, nanoparticle formulations, biocompatible carriers

## Abstract

This manuscript provides a comprehensive review of advancements in dry powder inhaler (DPI) technology for pulmonary and systemic drug delivery, focusing on proteins, peptides, nucleic acids, and small molecules. Innovations in spray-drying (SD), spray freeze-drying (SFD), and nanocarrier engineering have led to enhanced stability, bioactivity, and aerosol performance. Studies reveal the critical role of excipients, particle morphology, and device design in optimizing deposition and therapeutic efficacy. Applications include asthma, cystic fibrosis, tuberculosis (TB), and lung cancer, with emerging platforms such as ternary formulations and siRNA-loaded systems demonstrating significant clinical potential. Challenges such as stability, scalability, and patient adherence are addressed through novel strategies, including Quality by Design (QbD) approaches and advanced imaging tools. This work outlines pathways for future innovation in pulmonary drug delivery.

## 1. Introduction

DPIs are emerging as a transformative platform for the pulmonary delivery of therapeutic agents, especially synthetic biomolecules such as proteins, peptides, and nucleic acids. The pulmonary route offers unique advantages, including direct access to the lungs’ extensive surface area and vascular network, enabling localized treatment of respiratory conditions, and systemic delivery of biomolecules with high bioavailability. By bypassing the drawbacks of traditional parenteral routes—such as patient discomfort, risk of infection, and poor compliance—DPIs present a non-invasive, patient-friendly alternative for biomolecule administration [1,2,3,4].

Developing DPIs for synthetic biomolecules poses significant challenges due to the intrinsic sensitivity of these molecules to environmental conditions and mechanical stresses. For instance, proteins and peptides are highly susceptible to aggregation and denaturation during manufacturing, storage, and aerosolization. Similarly, nucleic acids like siRNA and mRNA require stabilization to maintain structural integrity and therapeutic activity. Advanced carrier systems, such as trehalose, mannitol, and lipid-polymer hybrids, have become essential for overcoming these barriers by improving stability, protecting biomolecules during processing, and enhancing aerosol performance [2,5,6,7].

The integration of DPIs with cutting-edge technologies—such as nanocarrier systems, SD, and particle engineering—has further expanded their utility for synthetic biomolecule delivery. Innovations like SFD and nano X-ray computed tomography (NanoXCT) enable precise control of particle size, density, and morphology, ensuring deep lung deposition and improved therapeutic outcomes. The ability of DPIs to achieve localized or systemic delivery makes them particularly effective in managing complex conditions, including respiratory infections, genetic disorders, and chronic inflammatory diseases [7,8,9,10].

Moreover, DPIs offer distinct advantages over other inhalation systems, such as pressurized metered-dose inhalers (pMDIs) and nebulizers. DPIs eliminate the need for propellants and external power sources, making them eco-friendly, portable, and cost-effective. Additionally, their dry formulations exhibit greater stability and lower susceptibility to microbial contamination compared to liquid-based systems. These attributes enhance patient adherence, reduce manufacturing complexities, and position DPIs as scalable solutions for global healthcare needs [11,12,13].

This review emphasizes the critical role of formulation science, carrier systems, and advanced processing techniques in optimizing DPIs for biomolecule delivery. This study highlights DPIs’ potential beyond respiratory applications, showcasing their transformative role in systemic therapies and precision medicine [1,2,4,14].

## 2. Overview of DPI Technology and Applications

### 2.1. Methods of Application for DPIs

DPIs are non-invasive drug delivery systems designed to target the respiratory tract, bypassing gastrointestinal degradation and hepatic first-pass metabolism. This makes them a vital alternative to oral and injectable therapies, particularly for sensitive biomolecules like peptides, proteins, and nucleic acids. DPIs ensure both systemic and localized therapeutic effects, thereby enhancing efficacy and patient comfort.

### 2.2. Pulmonary Delivery of Therapeutics

DPIs excel in delivering therapeutic agents directly to the lungs, achieving localized treatment with minimal systemic exposure. For instance, vasoactive intestinal peptide (VIP) derivatives, such as IK312532, have demonstrated enhanced anti-inflammatory efficacy in asthma and chronic obstructive pulmonary disease (COPD) models when delivered via DPIs with reduced systemic side effects [15,16,17,18]. Advanced DPI formulations have also effectively delivered siRNA and proteins, overcoming pulmonary barriers such as mucociliary clearance and alveolar macrophage activity [19,20,21]. Polyethyleneimine (PEI)-based siRNA carriers exemplify how DPIs facilitate targeted pulmonary gene delivery for localized treatment [19,21].

### 2.3. Advances in DPI Formulations

**Carrier-Based Systems for Enhanced Delivery:** Carriers such as lactose, mannitol, and trehalose are pivotal for optimizing the aerodynamic properties of DPI formulations. Innovations in nanoporous microparticles, engineered mannitol crystals, and needle-shaped carriers enhance fine particle deposition in deep lungs, maximizing therapeutic outcomes. Where lactose proves unsuitable, alternative carriers like sorbitol and xylitol demonstrate efficacy [22,23,24,25]. Ternary formulations combining active pharmaceutical ingredients (APIs) with excipients and fines further improve aerosol deposition in devices such as Rotahaler^®^ and Handihaler^®^ [26,27,28].

**Innovative Formulation Techniques:** Techniques like SD, freeze-drying, and high-shear mixing allow precise control of particle size, morphology, and aerodynamic properties, which are critical for effective lung deposition. For example, spray-dried budesonide/formoterol composites enhance FPF and dose uniformity, delivering superior therapeutic performance [22,29].

### 2.4. Device Engineering for Improved Performance

Modern inhaler designs, such as Diskus^®^, Turbuhaler^®^, and Easyhaler^®^, prioritize reliable dose delivery and minimal variability. Capsule-based systems like Twincer^®^ and Axahaler^®^ feature innovative designs to enhance patient adherence and drug dispersion. Resistance-optimized devices improve inspiratory flow control, ensuring better lung deposition [25,30,31,32]. Addressing common inhalation technique errors through patient training significantly enhances therapeutic outcomes, emphasizing the need for proper usage education [33,34].

### 2.5. Specialized Applications of DPIs

**Gene and Antimicrobial Therapy:** DPIs are instrumental in gene therapy, delivering siRNA and plasmid DNA with preserved bioactivity. Examples include spray-dried lipid nanoparticle (LNP) powders and PEGylated polymer carriers that target pulmonary tissues for effective gene silencing. Antimicrobial DPIs, such as colistin formulations, offer a convenient alternative to nebulizers for conditions like cystic fibrosis [19,20,32].

**Treatment of Chronic and Infectious Diseases:** Inhalable mannitol DPIs have shown benefits in cystic fibrosis by improving lung function and reducing exacerbation rates. Similarly, DPI formulations containing mometasone furoate and VIP derivatives provide targeted management of asthma and COPD [15,17,31].

**Protein and Peptide Delivery:** DPI innovations, such as spray-dried protein microparticles and peptide-loaded nanocarriers, preserve stability and bioactivity, enabling controlled release for systemic and localized effects. Examples include insulin-loaded DPIs and proliposomal formulations for TB therapy [35,36,37].

### 2.6. Innovations in Aerosol Performance

Advances in blending techniques and mixing strategies have significantly enhanced aerosol performance. High-shear mixing and ternary formulations improve dispersion and fine particle delivery. For instance, incorporating fines into lactose-based formulations optimizes drug detachment and deposition efficiencies [26,38]. Cutting-edge carriers, such as nano-in-microparticles and hybrid lipid-polymer systems, enable the co-delivery of multiple therapeutics, exemplifying the next generation of DPI technologies [39,40,41].

## 3. Therapeutic Potentials of DPIs for Synthetic Biomolecules

DPIs are increasingly being utilized for delivering protein-based therapies, where maintaining stability during aerosolization is critical. Proteins, prone to degradation, benefit from stabilizers like trehalose to preserve bioactivity and reduce aggregation. Techniques such as freeze-drying and nano-matrix particle engineering have significantly improved protein integrity and aerosol performance [42,43]. Examples include recombinant human growth hormone (rhGH) and ACE2 decoy polypeptides like LCB1, designed for targeted respiratory or systemic effects. These formulations offer non-invasive alternatives to injections, positioning DPIs as a promising vehicle for chronic diseases and rapid-response treatments for viral infections like severe acute respiratory syndrome coronavirus 2 (SARS-CoV-2) [44,45,46].

### 3.1. Asthma, COPD, and Respiratory Diseases

DPIs play a pivotal role in delivering treatments for asthma and COPD, including drugs such as budesonide, salbutamol, and formoterol. Achieving a high FPF is key for effective drug deposition in the lungs, facilitated by optimized particle blending techniques and the use of ternary mixtures [26,27]. For lactose-sensitive patients, alternative sugars like sorbitol and xylitol serve as safe carriers without compromising drug performance [47]. Combination inhalers, incorporating corticosteroids with bronchodilators like beclomethasone and formoterol, enhance therapeutic efficacy by reducing inflammation and expanding airways, effectively managing asthma symptoms and preventing exacerbations [48].

### 3.2. Antibiotic Delivery for Resistant Infections

Developing multi-drug DPIs for antibiotic delivery has gained attention for treating resistant lung infections. Co-SD combinations of antibiotics like meropenem, colistin, and rifampicin enhance aerosol performance and stability, targeting resistant Gram-negative bacteria effectively [49]. In conditions such as cystic fibrosis, where biofilm-associated pathogens are persistent, encapsulation methods prolong drug residence time and improve biofilm penetration, addressing limitations seen with traditional antibiotics [50].

### 3.3. Lung Cancer Therapy

In lung cancer treatment, DPIs are making strides with inhalable formulations of chemotherapeutic agents like doxorubicin (DOX) and docetaxel, sometimes paired with gene silencing agents such as ABCB1 shRNA. These therapies, delivered via nanoparticle carriers, focus on drug action within the lungs, boosting tumor targeting while minimizing systemic side effects [39,40]. The co-delivery of antioxidants such as genistein with chemotherapeutics enhances cellular uptake and mitigates oxidative stress in the tumor microenvironment, forming a comprehensive strategy against lung cancer [51].

### 3.4. Pulmonary Genetic Therapies and Biologics

Pulmonary delivery of genetic therapies, including siRNA and plasmid DNA (pDNA), targets disease-related genes within lung tissues. Formulations with nanoparticles composed of PEG, poly(lactide-*co*-glycolide) (PLGA), or PEI stabilize siRNA or pDNA, enhancing gene silencing and transfection efficiency while reducing toxicity [52,53]. siRNA targeting Vascular Endothelial Growth Factor (VEGF) has shown promise in suppressing tumor growth in lung cancer, while pDNA nanoparticles withstand the pulmonary environment, proving viable for treating respiratory diseases [53]. Proteins like alkaline phosphatase require stabilization during aerosolization to retain bioactivity, which can be achieved using additives like sodium carboxymethylcellulose (NaCMC) [54]. Bacteriophage platforms provide targeted treatment for multidrug-resistant (MDR) respiratory infections, supported by specialized devices and particle designs that maintain bioactivity during delivery [55,56]. This enhances the potential of inhalable biologics for addressing chronic and resistant respiratory infections.

### 3.5. Corticosteroids and Anti-Inflammatory Strategies

Inhalable corticosteroids, such as mometasone furoate and beclomethasone dipropionate, play an essential role in managing asthma and COPD by reducing inflammation and improving lung function. Administering mometasone furoate at night can improve lung function and reduce nocturnal asthma symptoms, enhancing patient quality of life [57,58]. Such targeted treatments aim to lower serum eosinophil cationic protein (sECP) levels, a biomarker of airway inflammation, to achieve improved symptom control.

### 3.6. Antiviral Therapies and Pandemic Preparedness

**Figure 1:**
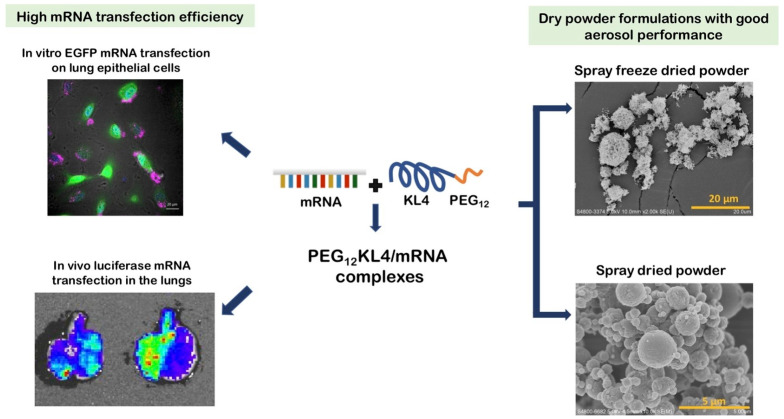
Efficient mRNA delivery and innovative dry powder formulations for advanced lung applications [60].

DPIs also have the potential to deliver antiviral siRNA and mRNA therapies to combat respiratory viruses, including influenza. Using pH-responsive carriers with stabilizers like mannitol enhances siRNA stability, targeting viral genes for effective silencing within lung cells [59]. Pulmonary mRNA vaccines offer a promising route for lung-specific gene expression, fostering localized immune responses against respiratory viruses while minimizing systemic exposure [60], an approach that could be pivotal in future pandemic preparedness. Figure 1 shows the development of PEG12KL4 as a novel mRNA delivery vector for pulmonary therapy. PEG12KL4/mRNA complexes demonstrated high transfection efficiency in lung epithelial cells and deep lung regions in vivo. These complexes were formulated into dry powders using SD and SFD, achieving excellent aerosol properties while preserving the biological activity [60].

### 3.7. Enzyme and Hormone Therapies for Chronic Conditions

Inhalable enzyme therapies, such as alpha-chymotrypsin and butyrylcholinesterase (BuChE), have been developed to treat inflammatory lung diseases and neutralize toxins. Electrospun fibers and PRINT particles enable sustained enzyme release, which is critical for prolonged action in the lungs [61,62]. BuChE, engineered for extended lung residence time, is valuable for rapid toxin neutralization and managing chronic pulmonary inflammation. Innovations in non-invasive hormone therapy for diabetes include inhalable insulin formulations, such as PLGA microcapsules stabilized with mannitol. These DPIs offer a patient-friendly alternative to injections, mimicking natural insulin release to enhance glucose control [36,63]. Advances aim to improve bioavailability and adherence, positioning inhalable insulin as a practical substitute for subcutaneous administration [64].

### 3.8. Polyphenolics, TB Therapies, and Other Applications

**Figure 2:**
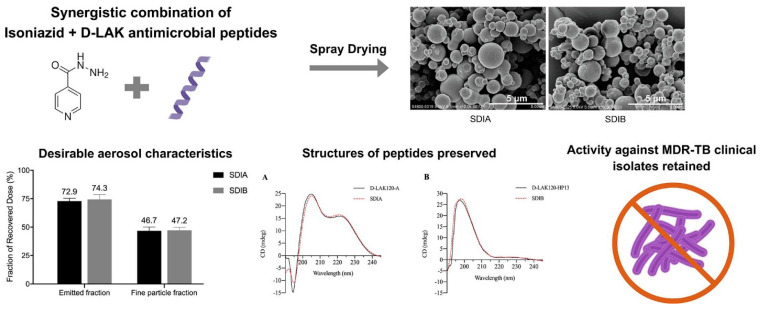
Synergistic INH + D-LAK dry powder formulation with preserved peptide structure, desirable aerosol properties (The section of structures of peptides preserved: Circular dichroism (CD) spectra of SDIA and SDIB formulations containing D-LAK120-A (**A**) and D-LAK120-HP13 (**B**). The unformulated peptides were included as controls for comparison) and retained activity against MDR-TB [66].

Polyphenolic compounds like curcumin, quercetin, and trans-resveratrol have been formulated as inhalable dry powders for treating corticosteroid-resistant asthma and COPD. Their anti-inflammatory properties make them suitable for patients who are less responsive to conventional treatments, offering complementary therapies that target inflammation directly without the side effects of corticosteroids [14]. DPIs contribute to the treatment of TB, particularly by combating MDR strains. Formulations that combine antitubercular agents like isoniazid (INH) with antimicrobial peptides in proliposomal dry powder forms stabilized with trehalose and L-leucine, enhance local antimycobacterial activity in the lungs [37,65,66]. Such DPIs deliver higher local drug concentrations, which are crucial for effectively managing MDR and extensively drug-resistant TB. Figure 2 illustrates the development of a novel inhalable dry powder formulation combining isoniazid (INH) and D-LAK antimicrobial peptides for tackling MDR-TB. This combination demonstrated synergistic antibacterial effects against MDR Mycobacterium TB clinical isolates, with optimal performance at a 2:1 mole ratio (INH:D-LAK). SD produced spherical particles with suitable aerodynamic properties for pulmonary delivery, retaining the peptides’ structure and antibacterial efficacy [66].

Table 1 highlights the various drug categories utilized in DPIs, their therapeutic purposes, and formulation strategies to enhance delivery. It provides insights into observed patterns, such as aerosolization efficiency, stability, and targeted delivery mechanisms, backed by scientific innovations like nanocarriers, ternary mixtures, and engineered particles. This table highlights the range of therapeutic applications of DPI systems, with a focus on targeted drug delivery and enhanced patient outcomes. Beta-agonists and corticosteroids are central to the treatment of asthma and COPD, with advancements in ternary systems and SD ensuring dose consistency and improved aerosolization. For infections, antibiotics formulated as engineered particles exhibit reduced systemic toxicity and improved stability. Pulmonary delivery of siRNA and mRNA via lipid-based or hybrid systems targets gene therapy and antiviral therapies effectively. Innovations like lactose-free formulations cater to pediatric and lactose-intolerant populations, while DPI insulin and anticancer agents demonstrate the potential of DPIs for non-respiratory conditions.

## 4. Carrier Materials: The Backbone of Effective DPI Delivery

Carrier materials play an integral role in the efficacy of DPIs by providing stabilization for active ingredients, enhancing dispersion, and optimizing pulmonary delivery. Trehalose is commonly used for stabilizing proteins and preventing dehydration and aggregation during aerosolization, which is crucial for maintaining bioactivity [1,42,54]. This ensures that the therapeutic efficacy is preserved for proteins administered via inhalation, making trehalose an effective choice for treating respiratory conditions or systemic protein delivery.

### 4.1. Enhancing Aerodynamic Properties and Stability

Fine lactose particles (also known as fines) are effective in improving the aerodynamic properties of DPI formulations, facilitating the dispersion of drugs such as corticosteroids and beta-agonists within the respiratory tract [11,22,26]. By enhancing flowability and the release profile, these carriers contribute to reliable therapeutic outcomes, especially in inhalation therapies that require precise pulmonary deposition. Additives like L-leucine and NaCl are also valuable in DPI formulations, enhancing the aerosolization of protein powders by reducing cohesive forces and promoting consistent drug delivery to the lungs [2,49,54]. For humidity-sensitive formulations, particularly those containing rSP-C, specialized carriers help maintain stability and effectiveness, even in moist environments, ensuring steady particle flow and preserving therapeutic function [75]. Advances in particle engineering have produced carriers like fine particle lactose (FPL) and micronized PEG, which enhance drug distribution and controlled release. These engineered carriers improve the FPF, facilitating disaggregation and consistent delivery to the targeted pulmonary regions [22,77].

### 4.2. Alternatives to Traditional Lactose Carriers

Alternatives to traditional lactose carriers, such as mannitol, sorbitol, and xylitol, provide enhanced stability and compatibility. Mannitol, in particular, offers better respirable fractions (RF%) and reduces carrier-drug adhesion, making it suitable for deep lung delivery of drugs sensitive to reducing sugars like corticosteroids [23,68,78]. Ternary mixtures that incorporate coarse and fine excipients like lactose have been shown to improve particle cohesion and dispersion, enhancing FPF and making them advantageous for asthma and COPD therapies [26,79]. Modified D-mannitol has been employed to optimize the physical properties of DPI formulations, including particle size, shape, and surface characteristics, leading to reduced drug retention and improved aerosolization [22,77]. Magnesium stearate is another carrier that enhances particle dispersion and stability, forming stable dry powder agglomerates that improve lung deposition. This has been particularly effective for drugs like melatonin and protein therapeutics that require consistent inhalation delivery [35,45,76].

### 4.3. Accommodating Sensitive Populations and Innovative Approaches

Lactose-free formulations are increasingly being developed to accommodate sensitive populations and avoid adverse reactions, broadening the accessibility of inhaler therapies [47,80]. Encapsulation using liposomal and polymeric microspheres offers sustained drug release and targeted retention, which are crucial for chronic respiratory conditions. Liposomal formulations have shown stabilization benefits and improved therapeutic efficacy in treating lung diseases [5,81]. Nanocarriers composed of materials like PEG, PLGA, and PEI enhance transfection efficiency and reduce toxicity, preserving nucleic acid integrity in the respiratory environment for pulmonary gene therapy [7,19,52,70]. Hybrid nanocarriers combining chemotherapeutic agents with gene therapy molecules provide innovative therapeutic approaches, utilizing lipid properties for controlled release and lung deposition, while minimizing systemic exposure. Cationic liposomes modified with dual peptides have been used to enhance cellular uptake and transport in gene therapy [39].

### 4.4. Advances in Particle Engineering for Sustained Release

Microparticle engineering has led to the development of particles with internal pore structures that support prolonged drug release, which is beneficial for chronic lung conditions requiring sustained therapeutic action. Porous microparticles based on PLGA extend the residence time in the lungs, increasing the efficacy of treatments for chronic illnesses, such as lung cancer and diabetes [36,73].

Table 2 presents the applications and observed outcomes of various carrier compositions and advanced formulation techniques for DPI systems. These entries describe their roles in enhancing drug delivery, bioavailability, and compatibility with diverse therapeutic agents. Supporting insights highlight the specific advantages and challenges associated with particle morphology, stability, and efficacy. The table outlines how carriers like lactose, mannitol, and trehalose play critical roles in DPI formulations by influencing particle formation, stability, and delivery efficiency. Lactose remains a standard carrier due to its compatibility with APIs and ability to optimize FPF. Mannitol, owing to its low hygroscopicity and enhanced aerosol performance, is emerging as a superior alternative. Hybrid systems and pH-responsive carriers facilitate the delivery of complex molecules like siRNA and peptides, addressing challenges like protein stability and targeted gene silencing. Advanced preparation techniques, including ternary mixtures and SD methods, have further improved drug dispersion and deep lung deposition.

## 5. Advanced Techniques for DPI Particle Formation

Manufacturing DPIs for synthetic biomolecules involves advanced techniques tailored to meet the stability, delivery, and aerodynamic needs of sensitive compounds. SD and SFD are the key methods for achieving precision in particle size and morphology control, which are essential for the development of fine particles suitable for inhalation. SD allows for the incorporation of stabilizers that protect fragile molecules from degradation, enhancing dispersibility, and retaining bioactivity. SFD, on the other hand, creates porous particles with a high surface area, improving aerosol performance and lung deposition [1,19,24,42,44,59,69,85].

### 5.1. Encapsulation and Stabilization Strategies

Nanoparticle and microparticle engineering, including the development of nano-embedded microparticles (NEM), enhances pulmonary delivery by facilitating controlled release and targeted deposition. Techniques such as emulsion/solvent diffusion and hybrid lipid-polymer encapsulation stabilize biomolecules and extend their residence time in the lungs. The use of liposomal encapsulation, often paired with SD, helps protect sensitive molecules and maintain their therapeutic efficacy from production through inhalation [7,8,20,50,52,70,81].

### 5.2. Carrier Blending and Controlled Release Mechanisms

Manufacturing techniques such as wet sieving, pearl milling, and spray congealing provide precise control over carrier-excipient blends. These methods ensure the production of particles with uniform dispersibility and size distribution, which are essential for effective lung deposition. Optimizing these processes enhances particle stability and aerosol performance, ensuring consistent dose delivery [6,35,77]. Specialized processing methods incorporating pH-responsive and cationic carriers have been used to achieve controlled release in various pH environments. Integrating materials such as PEI and ionizable cationic lipids stabilize biomolecules like siRNA and plasmid DNA during production, enhancing their protection against enzymatic degradation and ensuring precision in targeted pulmonary delivery. This has shown particular effectiveness in applications such as tumor-targeting VEGF-siRNA formulations [10,21,53,59,88].

### 5.3. Ensuring Stability and Performance Through Design

The QbD framework is essential for developing optimized DPI formulations, allowing for the systematic evaluation of critical parameters influencing aerosol performance. This approach ensures that DPIs meet stringent standards for FPF, dose uniformity, and stability, which are crucial for synthetic biomolecules that require precise bioavailability and consistent dosing [39]. Freeze-drying and lyophilization are employed to stabilize moisture-sensitive biomolecules, creating powders with an extended shelf life and consistent aerosol performance. These processes reduce moisture content and maintain stability during storage and transport, and lyophilized powders demonstrate improved aerosol properties, providing flexibility in storage conditions that preserve the functional integrity of biomolecules [52,81].

### 5.4. Innovative Engineering for Controlled Release

Layer-by-layer assembly and nano-to-micro engineering enable the precise control of biomolecule release kinetics and pulmonary targeting. Coating particles with layers of excipients results in stable formulations with customizable release profiles, protecting biomolecules from degradation. The production of “nano-in-micro” structures embeds biomolecules in micron-sized particles, facilitating sustained release and efficient lung deposition [40]. Co-crystallization offers a method for combining multiple drugs into stable multi-drug crystals that improve aerosol properties. This approach enhances solubility, bioavailability, and dispersibility by creating uniform crystal structures that stabilize each component. For synthetic biomolecules, co-crystallization supports the development of combination therapies with controlled delivery characteristics, preserving the structural integrity of each active ingredient [65].

### 5.5. Emerging Technologies for Precision Manufacturing

PRINT technology provides a scalable method for producing uniform particles with precise sizes and shapes, which is critical for consistent aerosolization and lung deposition. This technology allows for the creation of particles with predictable aerodynamic properties, which are essential for biomolecules that require controlled release and stability. The uniformity achieved enhances dose accuracy, ensuring that particles meet the deposition criteria needed for effective pulmonary therapies [9,62]. Proliposomal and hybrid lipid-polymer systems are produced through specialized techniques that allow liposome formation upon inhalation, providing a means of delivering poorly soluble biomolecules, including certain peptides and nucleic acids. These systems offer sustained release and protection against degradation, ensuring that DPIs are effectively converted in the lung environment and supporting the stability and absorption of biomolecules [37].

Table 3 reviews the various preparation and processing methods for DPI formulations, detailing their common applications, observed patterns, and the advantages they offer in terms of particle stability, delivery efficiency, and therapeutic efficacy. These supporting insights highlight the role of these methods in optimizing aerosol performance and enabling targeted drug delivery. Processing techniques are vital for shaping the efficacy and stability of DPI formulations. SD is extensively used to control particle size and morphology, enhancing FPF and bioactivity retention. SFD, while more specialized, supports sensitive molecules like proteins and siRNA by producing porous particles for deep lung deposition. Micronization ensures fine particles for direct aerosolization, and co-SD enhances stability by incorporating multiple agents in one step. Advanced particle engineering methods like PRINT and NanoXCT enable precise customization of particles for targeted therapies, while methods like ternary mixing address adhesion-cohesion balance for optimized drug dispersion and deposition.

**Figure 3:**
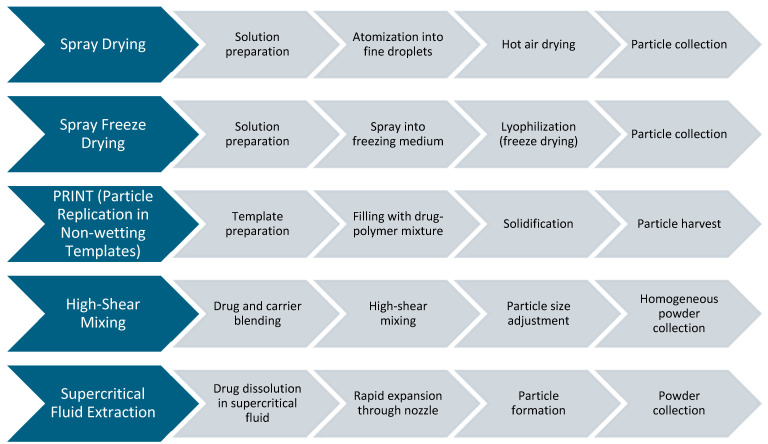
Overview of DPI Formulation Processing Techniques.

Figure 3 summarizes the five key processing methods for DPI formulations, each with unique advantages and challenges. SD offers scalability and precise particle size control but can result in low yield and thermal degradation of sensitive drugs. SFD produces porous particles with excellent dispersibility and is suitable for fragile compounds, though it is time-intensive and costly. PRINT technology enables precise shape and size control, enhancing drug delivery efficiency, but is limited by complex and expensive equipment. High-Shear Mixing is simple and cost-effective for blending carrier particles, but achieving uniformity in drug distribution can be challenging. Supercritical Fluid Extraction avoids high temperatures and solvents, preserving drug stability and purity, although it requires specialized equipment and may have limited scalability. These methods highlight the trade-offs between efficiency, cost, and product quality in the DPI formulation.

## 6. Physicochemical Characterization for Optimized DPI Delivery

Precise physicochemical characterization is fundamental for DPIs deliver synthetic biomolecules. Techniques such as Scanning Electron Microscopy (SEM) and laser diffraction ensure accurate particle size and uniform distribution, which are essential for effective drug deposition in the lungs [1,59,71,90]. Advanced methods like NanoXCT and Differential Scanning Calorimetry (DSC) provide further insights into particle structures that affect stability and bioavailability over time [8,37]. Comprehensive characterization facilitates the design of formulations with optimal aerodynamic properties, ensuring consistent and targeted pulmonary drug delivery.

### 6.1. Evaluating Aerosolization and Lung Deposition

A thorough evaluation of aerosolization and lung deposition is essential to optimize DPI performance and therapeutic efficacy. Key tests include:

**FPF:** FPF quantifies the proportion of particles that reach the lower respiratory tract, indicating the efficiency of drug delivery to the deep lung regions.

**Mass Median Aerodynamic Diameter (MMAD):** MMAD measures the median diameter of aerosolized particles, ensuring that their aerodynamic properties align with the deposition requirements for the lungs.

**Importance of Cascade Impactors:** Cascade impactors are commonly employed to analyze the aerodynamic particle size distribution, helping predict where particles will deposit in the respiratory tract [13,84,97]. Tests at varying flow rates simulate diverse patient inhalation patterns, enhancing device performance under real-world conditions.

**Reproducibility and Device Consistency:** Reproducibility testing ensures that DPIs consistently deliver accurate doses across multiple uses, which is critical for maintaining therapeutic outcomes.

**Integration of In Vitro and In Vivo Studies:** In vitro deposition studies using respiratory models are complemented by pharmacokinetic assessments in animal models. These combined approaches help predict drug distribution in the lungs, especially for formulations targeting deep lung tissues [6,28,41]. This multi-level evaluation is indispensable for ensuring the delivery efficiency of DPIs and minimizing off-target effects.

### 6.2. Stability Assessments and Long-Term Integrity

Ensuring the stability of DPI formulations under various environmental conditions is vital for maintaining drug efficacy during storage and use. Stability testing includes the following:

**Thermal and Physical Stability:** Techniques such as DSC and moisture content analysis monitor the thermal behavior and hygroscopicity of formulations.

**Real-World Stress Tests:** Storage under simulated real-world conditions (e.g., temperature and humidity fluctuations) evaluates the long-term integrity of DPIs, ensuring that they remain effective across the shelf life [61,83,88].

Stability assessments also involve cytotoxicity testing to confirm that the degradation products do not compromise patient safety. Aggregation analysis and bioavailability studies further verify the functional and structural integrity of the formulations, preventing loss of therapeutic performance over time [28,49].

### 6.3. Drug-Carrier Interactions and Encapsulation Efficiency

Drug-carrier interactions and encapsulation efficiency are pivotal for achieving sustained release and therapeutic effectiveness. Evaluation of these properties includes the following:

**Encapsulation Efficiency Testing:** This confirms the degree to which active biomolecules are protected within the carrier matrix, directly influencing drug release profiles.

**Release Kinetics:** Controlled release tests simulate inhalation conditions, evaluating how effectively drugs are released from carriers within the respiratory system.

**Drug-Carrier Compatibility:** Studies have assessed the stability of interactions between drugs and carriers to prevent premature drug release or aggregation, which could impair formulation consistency [5,81,87].

These evaluations provide insights into optimizing formulation design to improve patient outcomes.

### 6.4. Device Performance and Usability Testing

The performance and usability of inhaler devices are critical for ensuring efficient drug delivery. Tests specific to device functionality include the following:

**Peak Inspiratory Flow (PIF):** Simulating patient inhalation patterns, PIF tests determine whether patients can achieve sufficient flow rates for optimal drug release.

**Geometric Design Analysis:** Advanced imaging and flow studies optimize the device geometry to enhance aerosolization and delivery efficiency [30,93,98].

Comparative studies on DPIs and other inhalation devices have provided insights into usability, adherence, and patient preferences. Real-world usability assessments ensure that devices are user-friendly and meet patient needs, which is crucial for achieving consistent therapeutic outcomes [33,34,63].

### 6.5. Pharmacokinetics, Safety, and Bioactivity Evaluations

Pharmacokinetic, safety, and bioactivity evaluations are indispensable for understanding the therapeutic potential and safety of DPI-delivered drugs. Key tests include:

**Pharmacokinetic Studies:** These provide critical data on bioavailability, drug absorption, and clearance rates in the lungs, helping establish appropriate dosage levels [44,53].

**Cytotoxicity and Safety Testing:** Cytotoxicity assays evaluate the potential toxic effects of formulations on lung epithelial cells, while specific models assess safety in tumor suppression or infection control [73,99].

**Bioactivity Assessments:** These measure therapeutic effects, such as tumor growth suppression or infection resolution, offering insights into formulation efficacy and patient outcomes [37,74,100].

These evaluations guide formulation refinement and dosage optimization for specific respiratory conditions.

### 6.6. Gene Therapy-Specific Evaluations

For DPIs delivering gene silencing agents, such as siRNA, additional evaluations are necessary to confirm their therapeutic efficacy and minimize adverse immune responses.

**Transfection Efficiency:** This quantifies the ability of siRNA to penetrate target cells and suppress gene expression effectively.

**Cytokine Profiling:** Immune response tests, such as cytokine profiling, assess the risk of inflammation or immunogenicity, particularly in gene therapy applications [20,21,60].

**Gene Expression Changes:** Evaluations involving siRNA-loaded carriers measure changes in gene expression and assess inflammatory responses in lung tissue. These tests are vital for ensuring the safety and efficacy of gene therapy approaches [20].

### 6.7. Clinical Trials and Real-World Outcomes

Clinical trials remain the gold standard for evaluating the efficacy and safety of DPIs in real-world scenarios.

**Randomized Controlled Trials (RCTs):** RCTs assess lung function improvements, symptom reduction, and overall therapeutic outcomes in diverse patient populations [31,101].

**Patient-Centered Feedback:** Quality-of-life surveys and adherence studies provide insights into the practical usability of DPIs, informing refinements in device design and therapeutic regimens [48,58].

Long-term outcome evaluations further establish the role of DPIs in managing chronic respiratory conditions, ensuring that treatments meet patient needs effectively.

Table 4 outlines the various tests and analyses used to evaluate the DPI formulations, focusing on aerodynamic performance, stability, encapsulation efficiency, and therapeutic outcomes. Each method’s application is linked to improving drug delivery, ensuring consistency, and validating efficacy, with supporting insights into the tools and metrics employed. The table outlines the essential methods for evaluating DPI formulations to ensure their safety, efficacy, and consistency. Particle size and morphology analyses using SEM or laser diffraction ensure compatibility with inhalers and optimal lung deposition. Cascade impactor testing measures the aerodynamic particle size distribution (APSD) and FPF, which are crucial for achieving therapeutic efficacy. Stability tests identify factors like moisture sensitivity that can impact storage and use. Surface property analyses assess adhesion-cohesion interactions, aiding in formulation optimization. Additionally, encapsulation efficiency studies and in vivo deposition tests validate the performance of advanced therapeutics like siRNA and mRNA for targeted and systemic delivery.

## 7. Consistency of Bioavailability for Systemic Applications

Achieving consistent bioavailability is crucial for the systemic delivery of therapeutic agents via DPIs. By bypassing gastrointestinal degradation and hepatic first-pass metabolism, DPIs offer a reliable route for systemic drug delivery. Innovations in the formulation, device design, and integration have improved the ability of DPIs to deliver predictable therapeutic outcomes. Table 5 highlights the clinical applications and benefits of DPIs across a wide range of diseases, while Table 6 summarizes the key benefits of DPI systems, including their ability to enhance drug delivery efficiency, improve patient compliance, reduce systemic side effects, and offer long-term therapeutic and environmental advantages. Together, these tables highlight the transformative potential of DPIs in therapeutic applications.

### 7.1. Pulmonary Administration: A Reliable Gateway to Systemic Delivery

Pulmonary delivery through DPIs provides a direct route for drug absorption, avoiding the variability associated with oral or injectable administration. This approach ensures predictable systemic bioavailability and improves therapeutic consistency. Inhaled insulin products like Exubera^®^ and Afrezza^®^ exemplify this reliability, offering systemic delivery with improved patient compliance compared to traditional parenteral routes [3,4,42]. These systems have demonstrated consistent pharmacokinetics, highlighting the efficacy of pulmonary delivery in systemic therapies. As outlined in Table 5, DPIs are effective for non-invasive delivery in systemic diseases like diabetes [36,63,64], while Table 6 emphasizes how user-friendly designs improve adherence, particularly for pediatric and geriatric populations [13,33,67].

### 7.2. Encapsulation Technologies for Stability and Sustained Release

Encapsulation technologies, such as liposomes, PRINT nanoparticles, and proliposomal systems, have significantly improved systemic delivery by stabilizing drugs and enabling controlled release. PRINT nanoparticles, for instance, maintain protein integrity during aerosolization, ensuring reliable delivery. Similarly, proliposomal formulations like pretomanid DPIs have shown consistent systemic effects upon reconstitution, preserving the therapeutic activity of sensitive molecules [5,7,9,10,37]. These advancements are particularly beneficial for therapies targeting infections and resistant pathogens, as highlighted in Table 5 [37,49,50,56]. Additionally, Table 6 demonstrates the critical role of advanced excipients like mannitol and trehalose in enhancing formulation stability and improving aerosolization performance [1,2,24,35].

### 7.3. Moisture Resistance and Long-Term Stability

Formulations designed to resist moisture-induced degradation are essential for ensuring stable bioavailability over time. Moisture-resistant DPIs containing compounds such as Angiotensin [1,2,3,4,5,6,7], glycopeptide PNA5 powders, and anti-SARS-CoV-2 polypeptides have demonstrated retained plasma bioactivity and robust aerosol dispersion even under challenging storage conditions [14,44,46]. These advancements address one of the primary barriers to systemic delivery—ensuring stability during storage and transport. As shown in Table 6, stabilizers such as trehalose, mannitol, and raffinose enhance formulation durability and prevent degradation, contributing to long shelf-life stability [1,2,24,35].

### 7.4. Proteins and Peptides: A Systemic Alternative to Injectables

The inhalation route offers an effective alternative to the parenteral administration of proteins and peptides, ensuring consistent systemic bioavailability while reducing side effects. Stabilized vasoactive intestinal peptide (VIP) derivatives, for example, maintain chemical stability and therapeutic efficacy during pulmonary delivery [16,17,18,22]. Carrier-based DPIs incorporating excipients like leucine enhance pulmonary deposition and improve the reliable delivery of protein therapies. Table 5 highlights the application of these technologies in inflammatory lung diseases and systemic conditions [14,15,16,17], while Table 6 emphasizes DPI compatibility with advanced biologics, such as proteins and peptides, through innovative encapsulation approaches [5,7,60].

### 7.5. Gene Therapy: Ensuring Consistent Silencing and Targeting

Dry powder formulations for nucleic acid therapies have achieved remarkable consistency in their systemic effects. Lipid nanoparticle (LNP)-siRNA powders, for example, demonstrate over 90% protein downregulation with sustained gene silencing activity, establishing their efficacy in genetic therapies [21,52,85,88]. Similarly, plasmid DNA nanoparticles have shown stability during aerosolization and storage, demonstrating the reliability of DPIs for delivering genetic therapies with precision and consistency. As noted in Table 5, DPIs enable the effective delivery of siRNA, mRNA, and plasmid DNA therapies [7,19,52,70], while Table 6 highlights their ability to support the stability and bioavailability of complex molecules through advanced nanoencapsulation and hybrid systems [5,70].

### 7.6. Controlled and Prolonged Drug Release for Therapeutic Stability

Controlled and prolonged drug release is a critical factor in ensuring stable systemic bioavailability. DPI formulations, such as PAS-INH, for TB therapy provide synchronized dissolution, optimizing systemic drug concentrations [65]. Similarly, long-acting DOX microparticles sustain therapeutic concentrations for extended periods, highlighting the potential of DPIs for systemic cancer treatment [73]. As shown in Table 5, such innovations improve local deposition and treatment outcomes in diseases like TB and lung cancer [37,65,66]. Furthermore, Table 6 underscores the long-term clinical benefits of these formulations, including sustained improvements in pulmonary function and symptom control [31,57,101].

### 7.7. Alternative Carriers for Enhanced Consistency

Carriers play a vital role in the systemic consistency of DPIs. Mannitol, a non-lactose carrier, has emerged as a superior alternative for achieving stable pulmonary deposition and reliable systemic delivery. Its compatibility with sensitive biomolecules like proteins and peptides makes it a preferred choice for systemic DPI applications [72,86]. As highlighted in Table 5, mannitol-based carriers address the critical needs of pediatric asthma and lactose-intolerant populations [47,80,86]. Table 6 further emphasizes how alternative carriers like mannitol and trehalose enhance formulation flexibility and meet diverse therapeutic requirements [47,86].

### 7.8. Device-Formulation Integration: A Critical Component of Consistency

The interplay between device design and formulation significantly influences systemic bioavailability. Devices such as the Diskus inhaler deliver uniform doses across varying inspiratory flow rates, reducing the variability in therapeutic outcomes [67]. Pre-blending techniques, such as incorporating coarse lactose with active pharmaceutical ingredients, further minimize the variability in deposition and bioavailability [82]. These integrations are crucial for achieving predictable pharmacokinetics in systemic therapies, as demonstrated in Table 5 [11,29,33,48,67]. Moreover, Table 6 highlights the environmental and economic advantages of refillable, multi-dose DPI devices, which reduce treatment costs and carbon footprints [12,13,48].

### 7.9. Reliability in Cystic Fibrosis Therapies

In conditions requiring systemic delivery, such as cystic fibrosis, DPI innovations have demonstrated consistent therapeutic effects. The Twincer inhaler, for example, has achieved high adherence rates and stable outcomes in cystic fibrosis patients [32,71]. Spray-dried ternary formulations tailored for biofilm-targeted therapies offer stable release profiles, enhancing the reliability of systemic delivery under complex conditions. Table 5 highlights the application of DPIs in such therapies [32,71,91,101], while Table 6 emphasizes their efficacy in combating resistant infections and improving aerosolization through ternary antibiotic formulations [49,66,71].

### 7.10. Technological Advancements for Consistent Systemic Delivery

Advanced preparation techniques have been instrumental in ensuring precise pharmacokinetics and stable systemic delivery. Innovations, such as dual-peptide modifications and pH-responsive powders, have proven effective for gene therapy and biologics, reinforcing the role of DPIs in complex systemic applications [74,103]. As summarized in Table 5, these technologies enhance stability and therapeutic delivery for systemic diseases, including hormone deficiencies, gene therapies, and metabolic disorders [3,4,63]. Table 6 further highlights the rapid onset of action achieved through pulmonary delivery, which provides faster therapeutic effects in emergency situations and localized therapies [28,31,78].

Table 5 outlines the diverse clinical applications of DPI systems for respiratory and systemic diseases. It highlights the therapeutic advancements achieved through optimized DPI formulations, emphasizing patient benefits such as enhanced drug delivery efficiency, reduced side effects, and improved compliance. DPIs have been adapted for treating a diverse array of conditions, offering targeted and efficient drug delivery. In asthma and COPD, corticosteroids and beta-agonists delivered via DPIs achieve better FPF and dose consistency, reducing systemic side effects. Pulmonary delivery of antibiotics in cystic fibrosis improves lung clearance while minimizing systemic toxicity. For TB and lung cancer, nanoparticle formulations enable localized treatment, addressing resistance and minimizing toxicity. Emerging applications include siRNA and mRNA for genetic disorders and antiviral therapies. Pediatric and lactose-intolerant populations benefit from tailored formulations, while non-invasive insulin delivery exemplifies DPIs’ potential for systemic diseases.

Table 6 summarizes the key benefits of DPI systems, ranging from improved drug delivery and patient compliance to reduced systemic side effects and environmental advantages. It also highlights their versatility, stability, and efficacy in diverse therapeutic applications. DPIs significantly enhance therapeutic outcomes through their ability to deliver drugs with high precision and minimal systemic side effects. Advances in excipient engineering, including mannitol and trehalose, have improved stability and aerosolization performance. Their user-friendly design boosts compliance, especially in pediatric and geriatric populations. For complex drugs like siRNA and peptides, DPIs support bioavailability and stability through innovative encapsulation and hybrid systems. Environmentally friendly features and long-term clinical benefits, including sustained pulmonary function improvement and reduced treatment costs, further underscore their versatility and effectiveness across a wide range of diseases and patient populations.

## 8. Factors Influencing DPI Performance

### 8.1. Characterizing Particle Properties and Aerodynamic Performance

Particle characteristics, such as size, shape, density, and surface roughness, dictate aerosolization and lung deposition efficiency. Advanced analytical techniques, including laser diffraction, NanoXCT imaging, and SEM, are used to optimize these parameters. Performance evaluation using instruments like Andersen Cascade Impactor (ACI) and Next Generation Impactor (NGI) ensures that particles achieve an optimal MMAD and FPF for targeted delivery to the lower airways [6,7,43,93].

### 8.2. Balancing Extrathoracic and Intrathoracic Deposition Efficiency

Achieving the right balance between extrathoracic and intrathoracic deposition is critical for therapeutic success in aerosol delivery. The impaction parameter (da^2^Q) represents a critical determinant of aerosol deposition, particularly in balancing extrathoracic and intrathoracic delivery efficiencies. It is influenced by the particle’s aerodynamic diameter squared (da^2^) and volumetric flow rate (Q), which together define the deposition tendency of particles based on their size and velocity. Lower da^2^Q values favor deeper intrathoracic delivery, while higher values often lead to deposition in the extrathoracic region. Innovations in particle engineering, such as elongated mannitol crystals and ternary formulations with fine excipients, enhance drug dispersion and reduce adhesion forces, enabling higher FPFs and deeper lung deposition [30,76,78]. Advances in spray-drying techniques have further refined the particle morphology and aerodynamic properties, ensuring drug stability and efficient delivery [30,42,76,78,93].

### 8.3. In Vitro, Ex Vivo, and In Vivo Testing

To connect preclinical findings with clinical applications, a comprehensive approach that integrates in vitro, ex vivo, and in vivo testing methods is required. In vitro systems, such as cascade impactors and twin-stage impingers, assess aerosol performance metrics, while ex vivo techniques, like isolated perfused lung models, provide detailed drug-tissue interaction data. Complementary in vivo evaluations, including pharmacokinetic studies and imaging techniques, validate lung deposition patterns and systemic effects, ensuring the effective translation of aerosol therapies from laboratory to clinical settings [7,29,59].

### 8.4. Device-Specific Evaluations and Capsule Performance

The performance of DPI devices is tested to ensure optimal formulation compatibility. Parameters like inspiratory flow resistance, dose uniformity, and aerodynamic efficiency are critical. Capsule materials, including gelatin and HPMC, significantly impact powder flow, drug release, and aerosolization. Optimizing these factors improves the integration of devices and formulations [14,25,97].

### 8.5. Stability and Manufacturing Considerations

Stability testing evaluates the moisture content, aggregation, and glass transition temperature (Tg) to maintain particle integrity. Advanced manufacturing techniques, such as SD, freeze-drying, and high-shear blending, produce stable formulations. Carriers like lactose and mannitol are refined to enhance dispersion and resist humidity during storage [35,38,45,82].

### 8.6. Effect of Particle Size on Therapeutic Efficacy

Particles with aerodynamic diameters of 1–5 µm are optimal for reaching the lower respiratory tract. Specific formulations, such as cyclosporin A and polymeric microspheres, demonstrate enhanced respirable fractions (RF%) by optimizing the particle size. Particle engineering techniques like SD produce corrugated or needle-shaped particles that resist agglomeration and achieve higher FPFs [23,42,49].

### 8.7. Size Distribution of Inhaled Bio-Aerosols

A narrow size distribution ensures reproducibility and efficacy. Techniques like SD of budesonide/formoterol fumarate composites deliver consistent particle sizes, ensuring dose uniformity and therapeutic reliability. Structural features like corrugation and porosity reduce inter-particle cohesion, enhancing aerosol stability and dispersibility [2,8,29].

### 8.8. Internal Resistance of DPIs

Resistance to airflow in DPI devices directly influences the aerosolization efficiency. Devices like Novolizer^®^ manage resistance to maintain consistent FPFs across diverse patient groups. High-shear blending modulates resistance by balancing cohesive forces, ensuring effective dispersion, and reliable delivery [25,38,67,90]. Advances in formulations with excipients like L-leucine and cold-gelled HPMC capsules further optimize performance [25,37].

By integrating advanced engineering approaches, precise particle characterization, and optimized device design, DPI systems can achieve superior performance, ultimately enhancing therapeutic outcomes for patients with respiratory diseases.

## 9. Challenges and Future Directions in DPI Development for Biomolecules

### 9.1. Challenges in DPI Formulation and Delivery

Formulating DPIs for synthetic biomolecules, such as proteins, peptides, and nucleic acids, poses significant challenges due to the fragile nature of these therapeutic agents. Denaturation and aggregation during manufacturing, storage, or aerosolization can compromise bioavailability and therapeutic efficacy [1,42]. Achieving a consistent FPF and ensuring reliable pulmonary deposition are further complicated by the variability in mixing techniques, shear stresses, and environmental conditions, all of which impact aerosol performance and dosing accuracy [26,38]. Additionally, the hygroscopic nature of carriers like mannitol and glucose heightens moisture sensitivity, necessitating advanced stabilization strategies to mitigate degradation risks [6,47,86].

### 9.2. Patient Factors and Safety Concerns

Patient-related factors, such as the inhalation technique and inspiratory flow rates, critically influence the performance of DPIs. Variability in patient effort can significantly affect drug dispersion and deposition, particularly with passive DPIs [30,67]. While training programs can improve inhalation techniques, achieving consistent real-world usage remains challenging [33,34]. Moreover, extractables and leachables from device materials pose potential safety risks, necessitating rigorous testing and material selection to mitigate long-term health concerns [95].

### 9.3. Emerging Applications and Complexity in Formulation

The rise of therapeutic applications, such as gene and RNA delivery, introduces new complexities to DPI development. These biomolecules require carriers that protect their structural integrity during aerosolization, while ensuring effective pulmonary deposition and minimizing off-target effects. For instance, PLGA-based carriers have shown promise but may provoke mild inflammatory responses in sensitive populations [52,84]. To optimize therapeutic efficacy, excipient ratios must be carefully balanced and drying techniques refined to preserve stability and bioactivity [59,89].

### 9.4. Strategies for Minimizing Side Effects and Enhancing Safety

#### 9.4.1. Reducing Toxicity and Improving Stability

Systemic toxicity remains a significant concern, especially for sensitive molecules like peptides and proteins. Encapsulation technologies, such as liposomal carriers, stabilize drugs and reduce systemic exposure, as observed in cyclosporin A formulations [5,6]. Stabilizers like polysorbate 20 and zinc ions (Zn^2+^) prevent protein aggregation and degradation, ensuring safe and effective pulmonary delivery [7,10,45].

Nanocarrier systems bypass pulmonary barriers like mucociliary clearance, improving bioavailability and minimizing systemic side effects. Dual-targeted nanocomposites for cancer therapies, for example, localize drug action within the lungs, reducing off-target toxicity [51,62].

#### 9.4.2. Safer Anti-Inflammatory and Antimicrobial Therapies

Polyphenolic powders, as corticosteroid-sparing agents, provide anti-inflammatory benefits with fewer adverse effects, thus addressing the limitations of prolonged corticosteroid use [14,57]. Similarly, optimized DPI formulations for antibiotics and antivirals balance efficacy and safety. Moisture-resistant ternary antibiotic combinations preserve activity post-drying, reducing variability in dosing and associated side effects [44,49].

#### 9.4.3. Tailored Formulations for Vulnerable Populations

Ensuring safety in special populations, such as pregnant women and neonates, is a critical consideration. Tailored formulations like the Twincer DPI for cystic fibrosis patients demonstrate high adherence rates and minimal adverse effects, highlighting the need for population-specific solutions [32,104].

#### 9.4.4. Addressing Risks from Leachables and Extractables

Leachables and extractables from DPI devices pose toxicity and carcinogenicity risks. Comprehensive risk assessments and compatibility testing between formulations and device materials are crucial for regulatory compliance and patient safety [95].

### 9.5. Advancements in DPI Technology and Applications

#### 9.5.1. Innovations in Particle Engineering

Precision engineering techniques, such as PRINT, enable tailored particles with optimized aerodynamic properties [9,62]. Nanoparticles and nanosuspensions reduce particle cohesion, enhancing deposition efficiency and extending drug release [9,43]. Stabilizers, such as trehalose and hydrogel microspheres, have proven effective in preserving protein and peptide integrity [5,54].

#### 9.5.2. Advanced Carrier Systems

Hybrid carrier systems, integrating lipid-based nanoparticles with polymers like PEG and PLGA, combine stability with controlled release, reducing immune responses while improving therapeutic targeting [7,39,53]. Freeze-drying and moisture-buffering excipients enhance storage stability, widening the global applicability of DPIs [6,88].

#### 9.5.3. Intelligent Device Designs

Next-generation DPI devices with adaptive technologies address patient variability by enabling real-time adjustments to airflow resistance and powder release mechanisms. Such devices improve dosing consistency, particularly in pediatric and elderly populations [30,94]. Simplified interfaces and integrated feedback mechanisms further enhance patient adherence [13,33,34].

#### 9.5.4. Expanding the Therapeutic Scope

**Figure 4:**
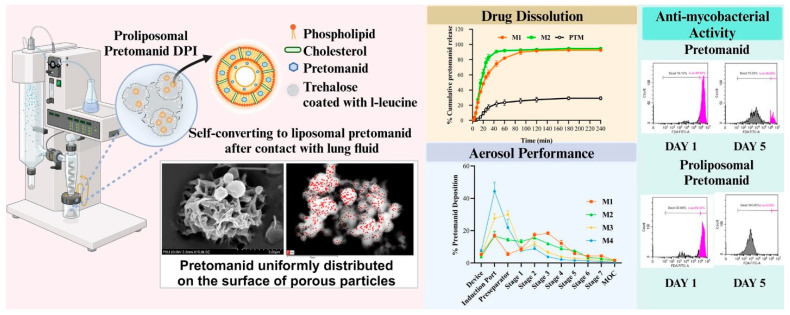
Proliposomal pretomanid DPI: Self-converting liposomal system with enhanced aerosol performance, drug dissolution, and antimycobacterial activity for pulmonary TB treatment [37].

DPIs are expanding beyond respiratory diseases to address multidrug-resistant infections, tuberculosis, and diabetes. For example, proliposomal systems for TB treatment combine L-leucine for aerosolization, achieving high drug content and favorable aerosol properties while maintaining safety for lung cells [37]. Figure 4 illustrates a proliposomal pretomanid DPI, showcasing its self-converting liposomal system with enhanced aerosol performance, improved drug dissolution, and potent antimycobacterial activity for pulmonary TB treatment [37].

Similarly, non-invasive insulin delivery via DPIs offers a promising alternative for diabetes management, mimicking subcutaneous injection pharmacokinetics while enhancing patient comfort [36,63].

## 10. Conclusions

DPIs are transforming the delivery of synthetic biomolecules, offering promising alternatives to invasive methods while enabling targeted treatments for diverse conditions. Significant advances in carrier systems, nanoparticle technology, and device engineering have led to improved therapeutic efficiency and patient compliance. However, challenges such as maintaining biomolecule stability, ensuring consistent dosing, and addressing patient technique variability remain critical. Progress in tailored carriers, intelligent device designs, and scalable manufacturing processes is essential for overcoming these barriers. The potential of DPIs to expand into systemic and chronic disease management underscores their importance in modern therapeutics, promising a future for safer, more effective, and patient-centric treatment modalities.

## Figures and Tables

**Table 1:** Therapeutic Applications and Advancements in DPI Drug Formulations.

Drug/Drug Category	Therapeutic Purpose	Observed Pattern	Supporting Insights	Refs. #
Beta-agonists (e.g., Salbutamol)	Asthma, COPD	Widely used in DPIs, often combined with lactose or mannitol carriers for improved aerosolization.	Finer particle carriers and ternary systems enhance performance; Diskus and MDIs show dose consistency.	[11,23,26,27,67]
Corticosteroids (e.g., Budesonide)	Asthma, Inflammatory lung conditions	Commonly paired with bronchodilators; ternary mixtures enhance fine particle delivery.	SD improves dose uniformity; alternative carriers like mannitol provide better respirable fractions.	[29,38,47,57,68]
Proteins and peptides (e.g., Lysozyme)	Pulmonary drug delivery, biopharmaceuticals	Stabilized using trehalose, mannitol, and SD; shows promise for systemic and local therapy.	Optimized using excipients like L-leucine and raffinose; protein stability is critical for therapeutic outcomes.	[1,2,4,24,46,69]
siRNA and DNA	Gene therapy, antiviral, antitumor	Nanocarriers and lipid-based formulations enable targeted delivery and high gene silencing efficiency.	siRNA-lipid complexes and pH-responsive peptides ensure deep lung deposition and therapeutic gene silencing.	[7,10,19,21,70]
Antibiotics (e.g., Colistin)	Bacterial lung infections	Engineered particles and ternary mixtures enhance stability and aerosolization for effective treatment.	Ternary antibiotic formulations with rifampicin improve hydrophobicity and reduce moisture sensitivity.	[32,49,50,71]
Insulin	Diabetes management	Alternative pulmonary delivery route being explored; challenges with bioavailability and patient adoption.	SD ensures particle size control; storage stability and clinical acceptability remain challenges.	[36,63,64]
Antimicrobial peptides (e.g., D-LAK120)	TB, bacterial lung infections	Formulated with mannitol carriers for sustained delivery and efficacy against resistant strains.	Synergistic combinations with INH and spray-dried powders show high potential for MDR-TB treatment.	[65,66,72]
VIP derivatives (e.g., IK312532)	Asthma, COPD, inflammatory airway diseases	Novel formulations show strong anti-inflammatory effects with efficient pulmonary deposition.	DPIs ensure rapid onset and prolonged receptor occupancy with minimal systemic side effects.	[15,16,17,18]
Polyphenols (e.g., Curcumin, Quercetin)	Anti-inflammatory, antioxidant therapy	Emerging as alternatives to corticosteroids for steroid-resistant patients or adjunct therapy.	Combination powders with resveratrol enhance anti-inflammatory effects; potential for safer long-term use.	[14,40,51]
Anticancer agents (e.g., DOX)	Lung cancer therapy	Nanoparticles and hybrid systems enable localized delivery and minimize systemic toxicity.	Hybrid lipid-protein nanoparticles and nano-in-microparticles show superior tumor targeting and efficacy.	[40,51,73,74]
Recombinant surfactants (e.g., recombinant surfactant protein-C (rSP-C))	Neonatal respiratory distress syndrome	Aerosolized proteins improve lung function in preterm neonates with precise particle size control.	Continuous aerosolization systems enable high-concentration protein delivery with minimal invasiveness.	[75]
mRNA	Gene therapy	Pulmonary delivery of mRNA shows promise for systemic therapies and vaccines.	PEG-based formulations ensure stability and transfection efficiency with no observed toxicity.	[60]
Melatonin	Sleep disorders, circadian rhythm management	Co-grinding techniques improve bioavailability and lung deposition for rapid therapeutic effects.	Magnesium stearate and lactose blends enhance aerosolization for effective DPI formulations.	[76]

**Table 2:** Carrier Systems and Formulation Strategies in Dry Powder Inhalation (DPI) Therapies.

Carrier Composition	Drug/Drug Category	Observed Pattern	Supporting Insights	Refs. #
Lactose (fine, micronized)	Various (Asthma, Beta-agonists)	Commonly used as a base carrier for DPI formulations.	Enhances FPFs; studied extensively for compatibility with multiple APIs.	[11,26,27,82]
Mannitol (spray-dried, engineered)	Various (Pulmonary drugs, Beta-agonists)	Increasing adoption as an alternative to lactose due to stability and particle size control.	Demonstrated improved respirable fraction and reduced hygroscopicity in ternary systems.	[23,31,47,68]
Trehalose and stabilizers (e.g., L-leucine)	Proteins, Peptides	Frequently used for protein stabilization in DPI formulations.	Improves protein stability during processing; supports high bioactivity retention and aerosolization.	[1,2,24,44,69]
Blends of coarse and fine excipients	Salbutamol, Ipratropium Bromide	Ternary blends to improve aerosolization and particle delivery efficiency.	Critical for competitive adhesion; studied for enhancing detachment and deposition in lower airways.	[26,27,82]
SD techniques	Protein and peptide therapeutics	Preferred method for producing DPI-compatible particles with stable morphology.	Yields FPFs with optimized flow properties; supports controlled release.	[24,42,46,83]
Polymeric carriers (PLGA, chitosan)	Gene Therapy, Chemotherapeutics	Utilized for encapsulation and controlled release in DPI systems.	Enhances biocompatibility and sustained release profiles; effective for advanced pulmonary therapies.	[39,52,84]
Lipid-based encapsulation systems	Proteins, siRNA	Emerging as an efficient carrier for stability and targeted delivery.	Supports systemic bioavailability and reduces systemic side effects and toxicity.	[5,51,85]
pH-responsive and nanocarrier systems	siRNA, DNA	Focused on gene silencing and nucleic acid delivery for advanced pulmonary therapies.	Improves transfection efficacy; enables targeted drug delivery for respiratory diseases.	[19,21,70]
Modified excipients (raffinose, glucose)	Various drugs	Use of non-traditional sugars as alternatives to lactose for specific patient needs.	Offers solutions for lactose-intolerant populations; maintains aerosol performance in humid conditions.	[16,80,86]
Hybrid systems (lipid-polymer)	siRNA, Anticancer agents	Utilized in advanced formulations to combine advantages of both lipid and polymer systems.	Achieves deep lung deposition, prolonged residence, and effective therapy against resistant diseases.	[39,40,74]
Ternary Mixtures (API-fine-coarse)	Multiple APIs	Improved FPF and deposition by optimizing carrier-API interactions.	Mixing order and excipient size critical to formulation success.	[26,27,82]
Engineered Particles	Proteins, Antibiotics	Tailored for optimized aerodynamic properties and stability.	Includes methods like SFD and PRINT for precise morphology and size control.	[1,50,87]

**Table 3:** Processing Techniques and Applications in DPI Formulations.

Preparation/Processing Method	Common Applications	Observed Patterns	Supporting Insights	Refs. #
SD	Proteins, peptides, antibiotics, corticosteroids	Most widely used for particle formation, providing control over particle size and morphology.	Enhances stability, FPF, and bioactivity retention; supports ternary mixtures.	[1,6,29,35,42,83]
SFD	siRNA, peptides, proteins	Produces porous particles with high aerosol performance and structural integrity for sensitive molecules.	Effective for siRNA, enabling deep lung delivery and high gene silencing efficiency.	[7,21,60,89]
Micronization	Amiloride, cetrorelix acetate	Effective for creating fine particles suitable for direct aerosolization or inclusion in adhesive mixtures.	Allows uniform drug dispersion in carriers; enhances deep lung penetration.	[70,90,91]
Ternary Mixing	Beta-agonists, corticosteroids	Combines coarse carriers, fine excipients, and APIs for optimized adhesion-cohesion balance.	Ternary mixtures with fine excipients (e.g., glucose, lactose) improve detachment and deposition profiles.	[26,27,79,82]
Nano SD	Lysozyme, proteins	Provides high precision for creating nanoscale particles for pulmonary drug delivery.	Achieves consistent particle sizes; Taguchi designs optimize stabilizer proportions for maximum bioactivity.	[2,24,44]
Co-SD	Antibiotics, protein-excipient combinations	Used to incorporate multiple drugs or stabilizers into a single particle, enhancing performance and stability.	Co-SD improves hydrophobicity, reducing moisture sensitivity in multi-drug formulations.	[46,49,81]
Wet Sieving and Spray Congealing	Mannitol, corticosteroids	Used for engineering particles with specific aerodynamic properties.	Spray-congealed particles outperform wet-sieved carriers for uniform dosing and lung deposition.	[77]
High-Shear Mixing	Budesonide, lactose blends	Optimizes blending of carriers and APIs to enhance aerodynamic properties and dosing uniformity.	Prolonged mixing times may increase cohesion forces; pre-blending with fines improves FPFs.	[28,38,78]
Particle Engineering (PRINT, NanoXCT)	Proteins, peptides, anticancer agents	Advanced methods for producing precision particles with defined structures for targeted delivery.	PRINT technology ensures uniformity and structural stability for therapeutic delivery to targeted sites.	[8,9,62]
Liposome and Nanoparticle Formulations	siRNA, anticancer agents, antibiotics	Liposomal systems enhance stability and enable sustained or targeted release of therapeutic agents.	Hybrid systems (lipid-polymer) combine the advantages of stability and controlled release for DPIs.	[39,74,81,92]
Recrystallization and Solvent Techniques	Mannitol, lactose, APIs	Engineered carriers like needle-shaped mannitol improve cohesion-adhesion and lung penetration.	Ethanol-water ratios create stable carriers; recrystallized particles show superior aerosolization properties.	[23,78]
Cascade Impactor Testing	Multiple DPI products	Essential for measuring aerodynamic particle size distribution (APSD) and ensuring formulation consistency.	Advanced CI methods reduce errors (e.g., bounce, re-entrainment) and improve regulatory accuracy.	[93,94,95,96]
Encapsulation Techniques (Liposome, PRINT)	siRNA, peptides, mRNA	Nanocarriers encapsulate fragile molecules to improve delivery and therapeutic efficacy.	Layer-by-layer techniques and hybrid nanocarriers enhance aerosol stability and targeting capabilities.	[7,39,74]
Humidity-Controlled Blending	Budesonide, alternative sugars (mannitol, sorbitol)	Conditioning carriers improve blend homogeneity and prevent degradation in humid conditions.	Non-lactose carriers such as mannitol exhibit lower hygroscopicity and better aerosolization profiles.	[6,28,47]
Co-grinding and Agglomeration	Melatonin, lactose	Co-grinding excipients improve blend homogeneity and aerosol performance for rapid drug release.	Magnesium stearate as an additive optimizes FPF and enhances delivery efficiency.	[76,78]
Multicomponent Dry Powders	Curcumin, ciprofloxacin, rifampicin	Complex combinations target biofilms, multi-drug resistance, and inflammatory conditions.	Ternary formulations address synergistic effects and enhance drug delivery in infection and inflammation.	[14,49,71]
pH-Responsive Systems	siRNA, plasmid DNA	pH-responsive peptides improve targeted nucleic acid delivery and transfection efficacy.	Enables efficient delivery to specific lung regions, ensuring therapeutic efficacy for respiratory diseases.	[19,59,70]

**Table 4:** Analytical and Evaluation Methods for DPI Formulations and Therapeutic Delivery.

Test/Analysis Method	Common Applications	Observed Patterns	Supporting Insights	Refs. #
Particle Size and Morphology Analysis	Proteins, peptides, antibiotics, corticosteroids	Essential for evaluating aerodynamic properties and compatibility with inhalers.	SEM, laser diffraction, and cascade impactors extensively measure particle size and distribution critical for lung deposition.	[1,22,24,35,89]
APSD, FPF	Multiple drugs	Key parameter for ensuring deep lung deposition and therapeutic efficacy.	Cascade impactors, Andersen samplers, and Next Generation Impactor (NGI) measure FPF and APSD; correlated with device design and formulation factors.	[6,93,96,99,102]
Bioactivity and Stability Testing	Proteins, peptides	Ensures therapeutic activity is retained during aerosolization and storage.	Circular dichroism, SEC, and stability under stress conditions assess protein stability and aggregation prevention.	[2,4,44,45]
Surface and Adhesion Properties	Beta-agonists, corticosteroids, proteins	Examines cohesion-adhesion balance for improved detachment and deposition.	Atomic force microscopy (AFM) quantifies interparticulate forces, which is critical for ternary mixtures and carrier-drug interactions.	[27,43,79,82]
Dosing Uniformity and Delivery Efficiency	DPI formulations	Critical for evaluating device performance and ensuring consistent drug delivery.	Fine particle dose (FPD) and dose uniformity assessed via multi-dose testing and factorial design studies.	[29,67,94,97]
In vitro Deposition Studies	Antibiotics, siRNA	Assesses deposition profiles in simulated respiratory tracts.	Twin impingers, cascade impactors, and anatomical inlets mimic lung deposition for clinical relevance.	[19,28,49,53]
Encapsulation Efficiency	Liposomal and nanoparticle formulations	Evaluates the loading capacity of carriers to optimize drug delivery and minimize waste.	Nanoencapsulation improves bioavailability; High-performance liquid chromatography (HPLC) and gel retardation assays measure drug loading efficacy.	[5,39,70,74]
Micromeritic and Flow Properties	Mannitol, lactose-based carriers	Ensures free-flowing powders suitable for inhalation devices.	Flowability, density, and Carr’s index measurements optimize blending and aerosolization.	[29,47,77,90]
Pharmacokinetics and Bioavailability	Insulin, siRNA, corticosteroids	Determines drug absorption, distribution, and therapeutic efficacy.	Animal models and clinical trials evaluate systemic absorption and therapeutic efficacy.	[53,63,64,88]
Stability Testing (Chemical and Physical)	All formulations	Ensures product integrity under storage and during use.	Accelerated stability testing highlights moisture and temperature sensitivities in DPIs.	[28,42,54,81]
Device Performance and Usability Studies	DPIs, MDIs	Examines inhaler design, reproducibility, and ease of use.	Expert validation and patient surveys identify usability issues and optimize inhaler designs.	[12,13,33,34]
In vitro Antibacterial/Antiviral Efficacy	Antibiotics, siRNA	Confirms activity against target pathogens for respiratory conditions.	MIC, biofilm inhibition, and antiviral activity assays validate formulation efficacy in infection models.	[49,59,66,71]
In vivo Lung Deposition and Efficacy	Anticancer agents, peptides	Evaluates therapeutic outcomes and tissue targeting in animal models.	Lung histology and broncheoalveolar lavage fluid (BALF) analyses confirm tissue targeting and therapeutic benefits.	[14,51,53,74]
Humidity and Environmental Sensitivity	Mannitol, lactose, alternative carriers	Evaluates the impact of relative humidity on formulation performance.	Carriers like mannitol exhibit lower hygroscopicity, which is crucial for storage stability and inhaler compatibility.	[6,47,86]
Antioxidant and Anti-inflammatory Testing	Polyphenols, VIP analogs	Confirms activity in reducing inflammation and oxidative stress.	Forced expiratory volume in 1 s (FEV1) improvement, granulocyte-macrophage colony-stimulating factor (GM-CSF) inhibition, and inflammatory marker analysis demonstrate efficacy in COPD and asthma.	[14,15,17,18]
Gene Silencing and Transfection Studies	siRNA, DNA, mRNA	Confirms therapeutic efficacy of nucleic acid delivery systems.	In vitro and in vivo gene knockdown and transfection assays validate pulmonary delivery potential.	[7,19,60,70]

**Table 5:** Clinical Applications and Benefits of DPIs Across Diseases.

Disease/Disorder	Intended Clinical Application	Key Insights and Proven Benefits	Refs. #
Asthma and COPD	Management of asthma and COPD using corticosteroids, beta-agonists, and bronchodilators.	Advanced DPI formulations improve FPF, reduce drug retention in devices, and enhance dose consistency.	[11,29,33,48,67]
Cystic Fibrosis	Pulmonary delivery of mucolytics, antibiotics (e.g., colistin), and anti-inflammatory agents.	Improved patient compliance with DPI devices like Twincer; mannitol enhances lung clearance and pulmonary function.	[32,71,91,101]
Lung Infections	Treatment of bacterial (e.g., Pseudomonas), viral, and fungal infections with inhaled antibiotics and peptides.	Multi-drug formulations improve stability and efficacy against resistant infections; aerosolized antibiotics reduce systemic exposure.	[37,49,50,56,66]
Inflammatory Lung Diseases	Reduction of airway inflammation using corticosteroids and anti-inflammatory agents like curcumin and VIP analogs.	DPI formulations deliver anti-inflammatory agents directly to affected regions, minimizing systemic side effects.	[14,15,16,17]
Diabetes	Pulmonary delivery of insulin as an alternative to subcutaneous injections.	Enhanced patient adherence due to non-invasive delivery; optimized particle engineering ensures consistent absorption.	[36,63,64]
Lung Cancer	Targeted therapy for lung tumors using chemotherapeutics, gene therapy, and siRNA.	DPI systems provide localized therapy with reduced systemic toxicity; nanoparticle formulations enhance lung retention.	[39,51,53,73]
TB	Pulmonary delivery of anti-TB agents for MDR-TB and XDR-TB.	Synergistic combinations of drugs improve local lung deposition and treatment outcomes; innovative carriers enhance stability.	[37,65,66,72]
Systemic Diseases	Pulmonary delivery of proteins, peptides, and nucleic acids for systemic therapeutic effects.	DPIs enable non-invasive delivery of sensitive molecules, improving bioavailability and reducing injection-associated risks.	[3,4,5,42,60]
Gene Therapy	Pulmonary delivery of siRNA, mRNA, and plasmid DNA for genetic and acquired lung diseases.	Effective for gene silencing and transfection, spray-drying technologies improve stability and therapeutic delivery.	[7,19,21,52,70,85]
Nocturnal Asthma	Targeting night-time asthma symptoms with extended-release corticosteroids.	Mometasone and related formulations improve night-time lung function and quality of life by reducing nocturnal symptoms.	[58]
Pediatric Asthma and Allergies	Management of asthma and allergy with lactose-free and hypoallergenic formulations.	Mannitol-based carriers ensure safety for lactose-intolerant and Cow’s Milk Protein Allergy (CMPA) patients, addressing a critical pediatric need.	[47,80,86]
Viral Infections	Treatment of respiratory viral infections with siRNA and antiviral agents like ACE2 decoys.	DPI formulations target lung-specific viral replication, reducing systemic effects and enhancing therapeutic efficacy.	[44,55,59,60]
Corticosteroid Resistance	Alternative therapies for corticosteroid-resistant asthma and COPD using polyphenols and VIP analogs.	Anti-inflammatory alternatives (e.g., curcumin) reduce inflammation without corticosteroid-associated side effects.	[14,16,17]
Respiratory Rare Diseases	Non-invasive treatment of rare respiratory conditions like neonatal respiratory distress syndrome.	rSP-C and similar therapies address gaps in neonatal care via pulmonary delivery systems.	[75]
Chronic Diseases	Pulmonary delivery for systemic management of chronic conditions, including hormone deficiencies and metabolic disorders.	Long-term studies confirm the stability and bioavailability of inhaled therapies for systemic applications.	[3,4,63]

**Table 6:** Proven Benefits of DPIs in Therapeutics.

Proven Benefit	Description	Supporting Insights	Refs. #
Enhanced Drug Delivery Efficiency	DPIs achieve higher FPF, improving deep lung deposition and therapeutic outcomes.	Optimized carrier-excipient combinations, ternary systems, and engineered particles enhance drug dispersion.	[11,27,29,49]
Patient Convenience and Compliance	Easy-to-use devices improve adherence, especially in pediatric and geriatric populations.	Multiple inhaler options (e.g., Diskus, Turbuhaler) are user-friendly and require minimal preparation.	[13,33,67]
Reduced Systemic Side Effects	Targeted pulmonary delivery minimizes systemic exposure, reducing side effects compared to oral or injectable routes.	Localized delivery of corticosteroids, siRNA, and antibiotics ensures concentrated action at the site of disease.	[59,71,82]
Stability and Shelf-life Improvements	Advances in SD, co-SD, and nano SD enhance formulation stability under varying conditions.	Stabilizers like trehalose, mannitol, and raffinose prevent the degradation and aggregation of sensitive molecules.	[1,2,24,35]
Versatility in Drug Classes	DPIs are effective for a wide range of therapeutic classes, including asthma, COPD, diabetes, and cancer.	Proven efficacy for beta-agonists, corticosteroids, proteins, siRNA, and antibiotics across multiple indications.	[4,14,26,74]
Environmental and Economic Benefits	DPIs are more eco-friendly compared to pMDIs, which use greenhouse propellants.	Refillable and multi-dose DPI devices reduce long-term costs and environmental impact.	[12,13,48]
Compatibility with Advanced Therapeutics	Suitable for delivering complex molecules like proteins, peptides, and nucleic acids.	Nanoencapsulation and hybrid systems enable the effective delivery of biologics and gene therapies.	[5,7,60,70]
Rapid Onset of Action	DPIs provide faster therapeutic effects due to direct drug delivery to the lungs.	Proven benefits in emergency asthma relief and localized antibiotic therapies.	[28,31,78]
Flexibility in Formulation Design	Compatible with diverse carriers, stabilizers, and excipients, allowing tailored formulations.	Mannitol, lactose, and alternative carriers offer flexibility to suit different drugs and patient needs.	[47,68,86]
Efficacy Against Resistant Pathogens	Ternary antibiotic formulations demonstrate superior aerosolization and activity against resistant infections.	Co-SD improves drug stability and enhances therapeutic action in challenging respiratory infections.	[49,66,71]
Improved Bioavailability	Pulmonary route bypasses first-pass metabolism, enhancing systemic bioavailability for select drugs.	Proven for insulin, siRNA, and other macromolecules in therapeutic trials.	[53,63,64,88]
Safety for Sensitive Populations	Lactose-free and hypoallergenic formulations ensure safety for lactose-intolerant and allergic patients.	Alternative carriers like mannitol and glucose provide viable solutions for these populations.	[47,80,86]
Long-term Clinical Benefits	Demonstrated sustained improvements in pulmonary function, symptom control, and quality of life.	Clinical trials confirm the long-term efficacy and safety of DPIs in asthma, COPD, and cystic fibrosis.	[31,32,57,101]
